# Contouring and augmentation of the temple using stromal vascular fraction gel grafting

**DOI:** 10.3389/fsurg.2022.893219

**Published:** 2022-08-16

**Authors:** Yuchen Zhang, Jialiang Zou, Yi Yuan, Jianhua Gao, Xihang Chen

**Affiliations:** Department of Plastic and Reconstructive Surgery, Nanfang Hospital, Southern Medical University, Guangzhou, China

**Keywords:** SVF-gel, fat grafting, volume retention rate, 3D scanning, temple

## Abstract

**Background:**

Hollowing temples are common in aging Asians. Stromal vascular fraction (SVF) gel is a novel, mechanically processed adipose-derived product containing condensed adipose-derived stem cells and native extracellular matrix, allowing improved fat grafting. The present study evaluated the effectiveness of SVF-gel treatment on temple hollowing.

**Methods:**

This prospective, single-center study included an SVF-gel grafting group (*n* = 34) and a Coleman's fat grafting group (*n* = 29). Temple contour was assessed using preoperative and postoperative photographs. Temple augmentation was quantified using three-dimensional (3D) technology and an MVS-600 3D scanner system. Patient satisfaction was assessed postoperatively.

**Results:**

At 12 months follow-up, the minimal forehead width/forehead width ratio and the width of the temporal peak were increased in both groups (*p* < 0.05).; and the retention rate (41.2% ± 8.4%) of the SVF-gel group was significantly higher than that of Coleman's fat group (32.6% ± 8.8%; *p* < 0.05). Moreover, patients in the SVF-gel group reported higher satisfaction scores than those in Coleman's fat group.

**Conclusions:**

SVF-gel is effective for temple contouring and augmentation., with increased efficacy compared with Coleman's fat.

## Introduction

The temple is a transitional subunit between the forehead and cheek, and temple hollowness is considered a typical characteristic of facial aging. Temple augmentation and facial contouring are commonly pursued aesthetic procedures in Asian patients.

Coleman's fat (autologous structural fat) grafting is a common and well-accepted technique for soft tissue contouring and volume enhancement, which was first introduced by Coleman in 1998. However, clinical observations and patient feedbacks have revealed that the long-term outcomes of Coleman's fat are diverse in some facial recipient sites, especially in the temporal region (1, 2). Thus, a more effective and reliable adipose-derived product is needed for clinical practice.

As reported previously, our department developed a novel adipose-derived product referred to as stromal vascular fraction (SVF) -gel (3), which is prepared mechanically and contains condensed adipose-derived stem cells (ASCs) and extracellular matrix (4–6). Most clinical studies and comparative animal experiments seem to demonstrate that the SVF-gel increased the retention rate of grafts at the long-term follow up (4–7).

The effects of SVF-gel on temple contouring and augmentation have not been fully assessed. In the present study, temporal SVF-gel and Coleman's fat grafting were tested in 63 patients. Temple contour analyses were collected and compared from preoperative and postoperative photographs by measuring cutaneous landmarks, and temple augmentation was quantified using three-dimensional (3D) technology and an MVS-600 3D scanner system (8, 9).

## Methods

### Patients and preoperative evaluation

All procedures involving human subjects were performed in accordance with the ethical standards of the Nanfang Hospital Ethics Committee and the 1964 Helsinki Declaration and its later amendments. Written informed consent was obtained from all patients.

From October 2018 to October 2020, a prospective, randomized clinical trial was conducted in the Department of Plastic and Reconstructive Surgery, Nanfang Hospital. Only individuals that underwent surgery for cosmetic purposes were included. Patients with topical or systemic diseases were excluded. Patients that could not undergo 3D scanning at the required time points were also excluded. Height, weight, and body mass index (BMI) were obtained. Patients were randomized into 2 groups: the SVF-gel group or Coleman's fat group. At the trial endpoint, 63 subjects had completed the follow-up and were included in statistical analyses. (see Supplementary SDC 1, which shows the flow diagram of subject enrollment and outcomes.)

### Surgical techniques

#### Fat graft harvesting and processing

**Figure 1:**
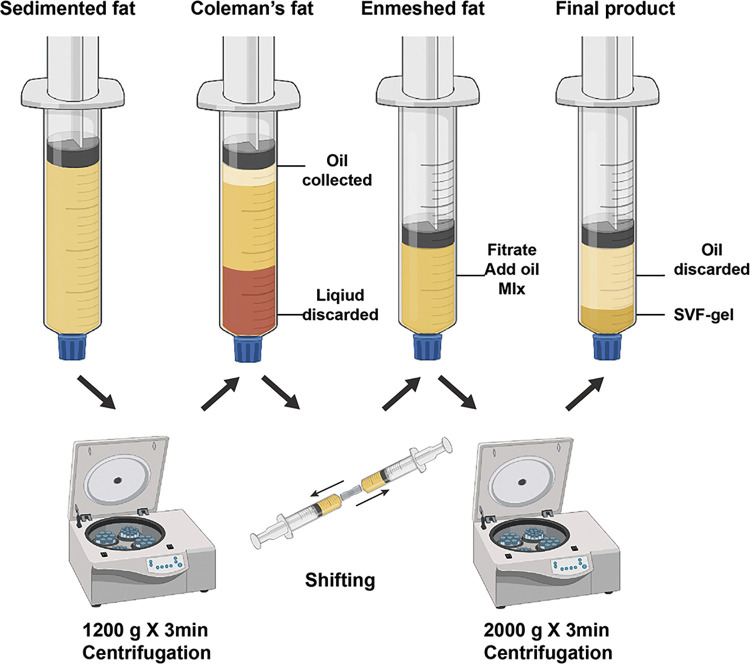
Processing procedure for SVF-gel.

With the patient under general anesthesia, subcutaneous adipose tissue was harvested from the abdomen or thigh. After harvesting, lipoaspirates were centrifuged at 1,200 × g for 3 min to obtain Coleman's fat. SVF-gel was prepared as previously reported (3). Briefly, standard Coleman's fat was mechanically emulsified by shifting between two 10-ml syringes connected by a female-to-female Luer-Lok connector with an internal diameter of 2.4 mm. The shifting speed remained stable at 10 ml/second for 1 min. The emulsified suspension was then processed by centrifugation at 2,000 × g for 3 min. The middle layer, which presented a sticky substance, was collected as SVF-gel (Figure 1).

#### Injection of fat grafts

**Figure 2:**
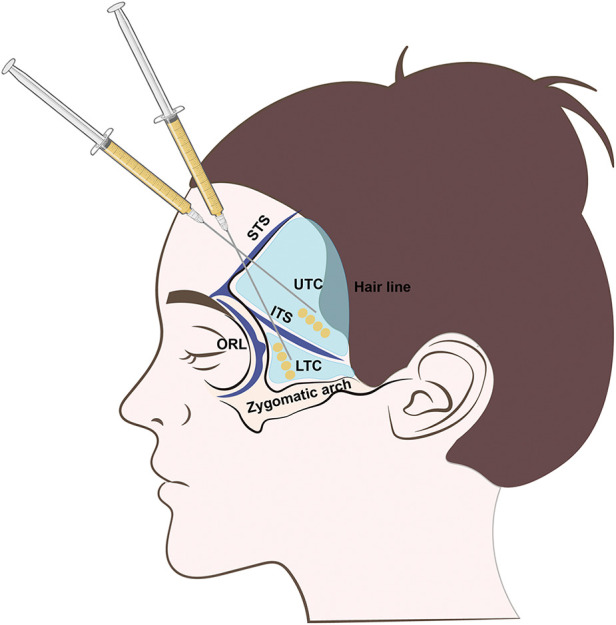
Illustration of temple fat grafting. LOFC, lateral orbital fat compartment; UTC, upper temporal compartment; LTC, lower temporal compartment; STS, superior temporal septum; ITS, inferior temporal septum; ORL, orbital retaining ligament; ZCL, zygomatic cutaneous ligament.

Prepared SVF-gel and Coleman's fat were transferred to 1 ml Luer-Lok syringes respectively for injection. A small incision was made at the middle of the temporal line. Lidocaine (1%) with 1:100,000 epinephrine was injected into the loose areolar tissue/parotid-temporal fascia of the temple. Subsequently, an 18 G single-holed, blunt-tipped infiltration cannula was inserted into this layer. Within this layer, the fat graft was placed in the upper and lower temporal compartments using a multiplane, multi-tunneling technique (Figure 2). Injected graft volumes were recorded in milliliters. No subjects received additional surgical interventions to the face during the trial.

### Outcome evaluation

#### Temple contour analysis

Standardized photographs were collected using a Canon EOS 70D camera (Canon, Inc., Tokyo, Japan). Preoperative photographs were taken before surgery, and postoperative photographs were taken at 1, 6, and 12 months follow-ups.

**Figure 3:**
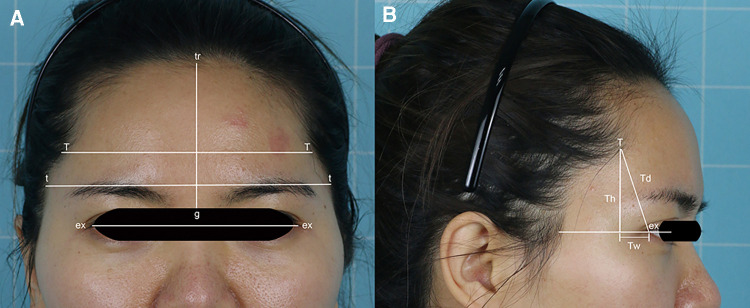
Measurements of the temporal hairline. ex-ex, intercanthal (ex- ex); Td, temporal peak depth; Th, temporal peak height; tr-g, forehead height; t-t, forehead width; T-T, minimal forehead width; Tw, temporal peak width.

Data measurement was performed in the frontal and lateral views of preoperative and postoperative photographs. All measurements were collected using Adobe Photoshop CC 2019 (Adobe Systems, Inc., San Jose, CA). The following anthropometric measurements were evaluated according to Diao et al. (10), as summarized in Figure 3.

#### Temple augmentation analysis

Three-dimensional imaging was performed to assess subjects preoperatively and at 1, 6, and 12 months follow-ups using an MVS-600 3D scanner system (Zhongkezhimei Technology Co., Ltd., Shenzhen, People's Republic of China). Subjects were seated approximately 80 cm from the front of the scanner, and facial images were captured in 2 s (see Supplementary SDC 2, Figure, which shows a schematic of the 3D imaging). Three-dimensional models were automatically generated using a CASZM software system after approximately 10 s of scanning, generating a colored hypsography. The temporal hollowing region was marked by four borders according to a previous study (11): Superiorly, the superior temporal line; Anteriorly, the lateral orbital rim; Inferiorly, the superior border of the zygomatic arch; Laterally, the temporal hairline. The volume discrepancy of the landmarks of the temple between preoperative and postoperative 3D images was calculated. Graft retention rates were calculated as (retention volume / injection volume) × 100%.

#### Patient 5-point satisfaction scale

All patients were asked to complete a satisfaction survey to rate their level of satisfaction with the treatment results at the 12 months follow-up visit. The 5-point scale was graded as (1) very dissatisfied, (2) dissatisfied, (3) neither, (4) satisfied, or (5) very satisfied.

### Statistical analysis

Statistical analyses were performed by an independent researcher using IBM SPSS Version 24.0 software (IBM Corp., Armonk, NY, USA), and all the results are expressed as mean ± standard deviation (SD). Baseline characteristics and non-parametric outcomes between the two study groups were compared using a related-samples Wilcoxon signed rank test, and two-tailed Student's t-tests were performed to test continuous variable outcomes. In all analyses, statistical significance was defined as *p* < 0.05.

## Results

### Patient characteristics

Of the 63 patients, 34 subjects were in the SVF-gel group and 29 subjects in Coleman's fat group. No significant differences in sex, age, or BMI were found between both groups (*p* > 0.05). Patient BMI was reassessed at the 12 months follow-up, and no significant intergroup differences were detected (*p* > 0.05) (see Supplementary SDC 3, Table, which summarizes subject demographics). Furthermore, there was no statistical significance in the percentage of donor sites between groups (*p* > 0.05) (see Supplementary SDC 4, Table, which summarizes fat harvesting sites and injection volumes). Transplant procedures were successful in all subjects.

### Improved temple contours in both groups

In the SVF-gel group, the T-T to t-t ratio increased from 0.87 ± 0.02 to 0.89 ± 0.03 (*p* < 0.05). This indicated an increase in temporal breadth. Normalized Tw slightly increased from 0.19 ± 0.06 to 0.23 ± 0.05 (*p* < 0.05). In Coleman's fat group, there was no statistically significant difference between the T-T to t-t ratio pre-and postoperatively (0.86 ± 0.05 to 0.90 ± 0.02 (*p* < 0.05). Normalized Tw modestly increased from 0.2 ± 0.07 to 0.24 ± 0.07 (*p* < 0.05). These findings indicated that the temporal peak shifted slightly outward in both groups after treatment, and there were no statistically significant differences between the two groups. All data are shown in Table 1.

**Table 1:** Measurements of the temporal hairline.

		SVF-gel group			Coleman's fat group			
Measurement	Normalized measurement	Preoperative Mean (SD)	Postoperative Mean (SD)	*p*1	Preoperative Mean (SD)	Postoperative Mean (SD)	*p*2	*p*3
Minimal forehead width/forehead width	(T-T)/(t-t)	0.87(0.02)	0.89(0.03)	<0.05	0.86(0.05)	0.90(0.02)	<0.05	0.13
Temporal peak width	Tw/(ex-ex)	0.19(0.06)	0.23(0.05)	<0.05	0. 2(0.07)	0.24(0.07)	<0.05	0.51
Temporal peak depth	Td/(tr-g)	0.61(0.09)	0.65(0.09)	0.07	0.64(0.09)	0.68(0.09)	0.09	0.19
Temporal peak height	Th/(tr-g)	0.54(0.07)	0.56(0.07)	0.24	0.55(0.10)	0.57(0.09)	0.42	0.62

Abbreviations: ex, exocanthion; g, glabella; *p*, *p*-value; SD, standard deviation; T, T point; t, temporal point; Td, temporal peak depth; Th, temporal peak height; tr, trichion; Tw, temporal peak width.

Values are expressed as mean (standard deviation). A two-tailed Student's t-test was used to generate p-values.

*p*1 denotes the comparison between pre- and postoperation in the SVF-gel group.

*p*2 denotes the comparison between pre- and postoperation in the Coleman's fat group.

*p*3 denotes the comparison between the SVF-gel and Coleman's fat groups postoperatively.

### Increased SVF-gel retention relative to conventional fat grafts

**Figure 4:**
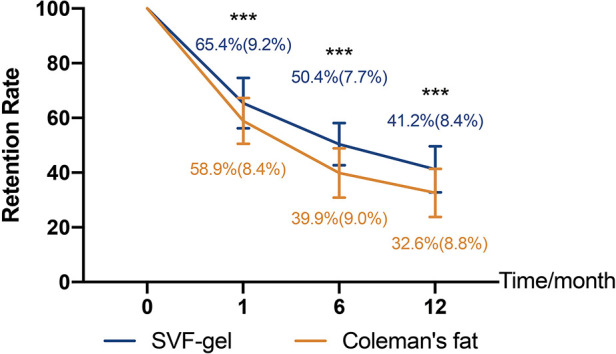
The retention rates of SVF-gel and Coleman's fat at 1, 6, and 12 months follow-ups.

The average injected volume was 5.5 ± 1.0 ml in the SVF-gel group and 5.9 ± 1.4 ml in Coleman's fat group (*p* > 0.05). At the 1 month follow-up, the retention rate of grafts in Coleman's fat group was statistically lower than that in the SVF-gel group (58.9% ± 8.4% and 65.4% ± 9.2%, *p* < 0.05). At the 6 months follow-up, retention rate was higher in the SVF-gel group (50.4% ± 7.7% and 39.9% ± 9.0%, *p* < 0.05). Consistently, at the 12 months follow-up, the retention rate was higher in the SVF-gel group (41.2% ± 8.4% and 32.6% ± 8.8%, *p* < 0.05). The retention volumes and rates are shown in Table 2 and Figure 4.

**Table 2:** Retention rates of SVF-gel and Coleman's fat injection at 1, 6, and 12 months follow-ups.

Retention	SVF-gel group		Coleman's fat group		*p*-value	
Time, months	Volume, ml	Rate	Volume, ml	Rate	Volume, ml	Rate
1	3.6(1.0)	65.4% (9.2%)	3.5(1.1)	58.9% (8.4%)	0.59	<0.01
6	2.8(0.7)	50.4% (7.7%)	2.3(0.8)	39.9% (9.0%)	<0.01	<0.01
12	2.3(0.7)	41.2% (8.4%)	1.9(0.7)	32.6% (8.8%)	<0.01	<0.01

Values are expressed as mean (standard deviation). A two-tailed Student's t-test was used to generate *p*-values.

### Patient satisfaction

At the 12 months follow-up, higher satisfaction scores in the SVF-gel group than that in Coleman's fat group. 79.4% of subjects reported high satisfaction rates (rated 4 or 5) in the SVF-gel group, and 62.0% of subjects had high satisfaction rates (4 or 5), with 7.0% of subjects reporting to be dissatisfied (1 or 2) in Coleman's fat group (Table 3).

**Table 3:** Patient satisfaction rates of patients in the SVF-gel group and Coleman's fat groups.

	Very unsatisfied (*n*, %)	Unsatisfied (*n*, %)	Neutral (*n*, %)	Satisfied (*n*, %)	Very satisfied (*n*, %)	*p*-value
SVF-gel group	0	0	7 (20.6%)	21 (61.8%)	6 (17.6%)	<0.05
Coleman's fat group	0	2 (7.0%)	9 (31.0%)	15 (51.7%)	3 (10.3%)	<0.05

A Wilcoxon signed rank test was used to generate *p*-values.

### Case demonstrations

#### Case 1

**Figure 5:**
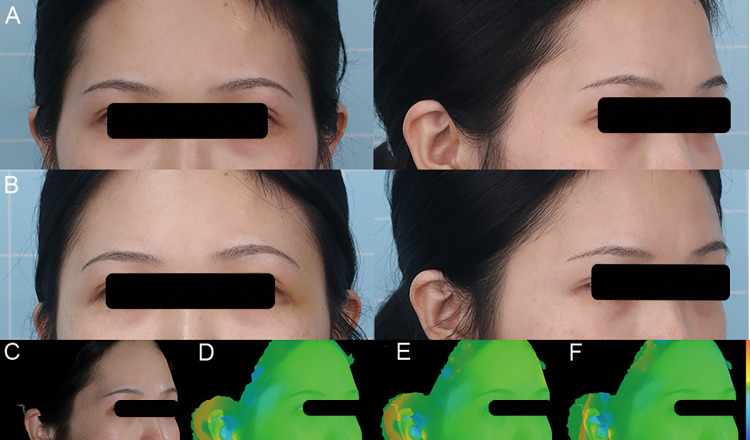
A 28-year-old female patient presented with temporal hollowness and a prominent tear trough deformity and was treated with one SVF-gel grafting procedure. (**A**) Preoperative front and right oblique photos. (**B**) Preoperative front and right oblique photos at 12 months. (**C**) Preoperative right oblique photo. (**D–F**) 3D reconstruction of right oblique images at 1, 6, and 12 months.

A 28 years old female patient presented with temporal hollowness. SVF-gel (5.8 ml) was injected into the right temple, and 6.3 ml was injected into the left side. Preoperative photography and postoperative 1, 6, and 12 months follow-up photography are presented. Temporal hollowness was improved (Figure 5).

#### Case 2

**Figure 6:**
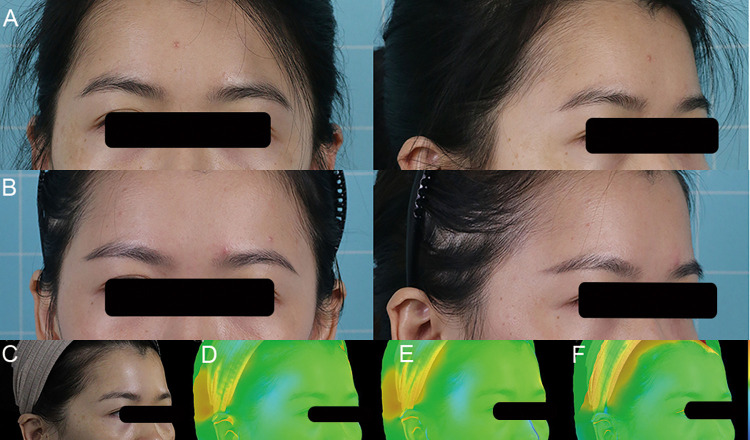
A 23-year-old female patient presented with temple hollowing and was treated with Coleman's fat grafting. (**A**) Preoperative front and right oblique photos. (**B**) Preoperative front and right oblique photos at 12 months after one Coleman's fat grafting procedure. (**C**) Preoperative right oblique photo. (**D–F**) 3D reconstruction of right oblique images at 1, 6, and 12 months.

A 23-year-old female patient with the moderate temporal hollowness of both temples underwent one autologous fat grafting procedure. A total of 12 ml Coleman's fat was grafted in both temples. Twelve months after the grafting treatment, the patient appeared to have no hollowness in both temples (Figure 6).

#### Case 3

**Figure 7:**
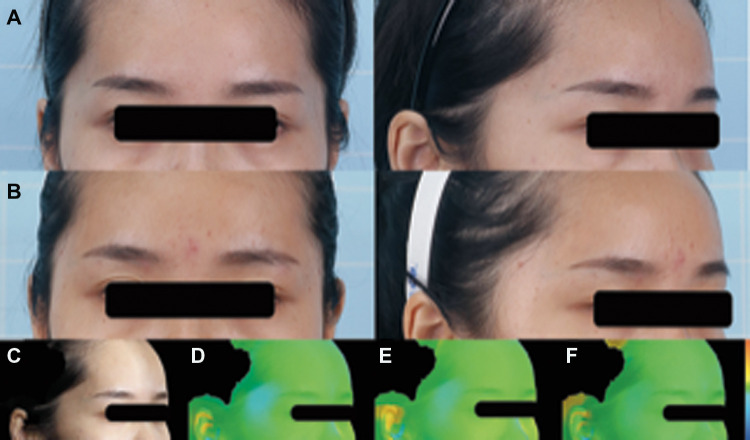
A 27-year-old female patient complained about temporal hollowness on both sides. SVF-gel (3.9 ml) was injected into the right temple, and 4.3 ml was injected into the left temple under one autologous fat grafting procedure. No temple hollowness was found at 12 months in either temple. (**A**) Preoperative front and right oblique photos. (**B**) Preoperative front and right oblique photos at 12 months. (**C**) Preoperative right oblique photo. (**D–F**) 3D reconstruction of right oblique images at 1, 6, and 12 months.

A 27-year-old female patient complained about temporal hollowness on both sides. SVF-gel (3.9 ml) was injected into the right temple, and 4.3 ml was injected into the left temple underwent one autologous fat grafting procedure. No temple hollowness was found at 12 months in either temple (Figure 7)

#### Case 4

**Figure 8:**
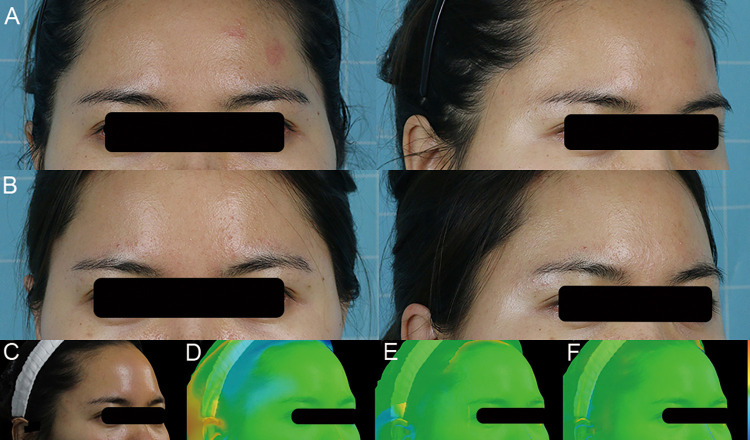
A 31-year-old female patient presented with temporal hollowness. A total of 8 ml of Coleman's fat was injected into both temples. Temporal hollowness was improved at the 12 months follow-up. (**A**) Preoperative front and right oblique photos. (**B**) Preoperative front and right oblique photos at 12 months. (**C**) Preoperative right oblique photo. (**D–F**) 3D reconstruction of right oblique images at 1, 6, and 12 months.

A 31-year-old female patient presented with temporal hollowness. A total of 8 ml Coleman's fat was injected into both temples. Temporal hollowness was improved at the 12 months follow-up (Figure 8).

## Disscusion

This study reports the results of a prospective, randomized, clinical trial designed to assess the temple contouring and augmentation effects of SVF-gel in comparison with conventional Coleman's fat grafting. The minimal forehead width/forehead width ratio was increased in both groups postoperatively, as well as the width of the temporal peak. In addition, at 12 months postoperatively, the SVF-gel retention rate was significantly higher than that of Coleman's fat grafts, and patients reported a higher clinical satisfaction score in the SVF-gel group than in Coleman's fat graft. These findings suggest that SVF-gel can be considered an ideal filler for temple contouring and augmentation, and could present advantages over conventional fat grafting.

Fat grafting and other filler injections have been widely applied in facial contouring for decades. However, the selected techniques are based on surgeons' clinical experiences, and the injection plane for filler placement varies between surgeons (12). An anatomical study of the temple (13) showed that the temporal region is comprised of two compartments, the UTC and the LTC, which are separated by the ITC. Tissue layers in the temple consist of layer 1, dermis; layer 2, subcutaneous layer; layer 3, superficial temporal fascia; layer 4, loose areolar tissue / parotid-temporal fascia; and layer 5, deep temporal fascia (13, 14). Layer 3, the superficial temporal fascia, is highly vascularized, containing the frontal branch of the superficial temporal artery and the sentinel vein (14). Therefore, we did not place the fat grafts into layer 3. Contrastingly, layer 4, the loose areolar tissue/parotid-temporal fascia of the temple, encompasses one sentinel vein and several perforator vessels but is otherwise predominately avascular (15, 16), so it was considered to be an optimal injection site. Prior studies of temporal hollowing augmentation suggested placing fat grafts into both layer 2 and layer 4 (10, 14). This strategy can be effective, but novice surgeons could have difficulty in precisely injecting fat grafts into layer 2. Therefore, we chose layer 4 as the fat graft injection layer in the present study, which is a safe and effective recipient site for temporal fat grafting.

Coleman's fat grafting has been commonly used in facial contouring and augmentation. However, no standard for temple aesthetics exists. The objectives of temple contouring should include the following: to restore a gentle, slightly convex curvature from the zygomatic process upward toward the forehead (17, 18), to increase the minimal forehead width (10, 17), and to correct inwardly curved temporal hairlines (10). In the present study, we observed that both Coleman's fat and SVF-gel grafting satisfactorily smoothened the temporal contour and increased the minimal forehead width and the width of the temporal peak.

In addition, graft volume change was quantitatively measured. Three-dimensional imaging was performed using a CASZM MVS-600 with an accuracy of up to 0.05 mm, which is sufficient for volumetric analysis. Moreover, 3D image reconstruction and volume differences with gradual changing of colors were performed, providing an accurate and intuitive image of retention volume and therapeutic effects. Three-dimensional visualization of pre-and post-operative images and changes was used to identify the precise filling location and volume, and to evaluate the effectiveness of fat grafting treatment.

Patients in both groups underwent 3D scanning preoperatively and at 1, 6, and 12 months postoperatively. The results of data demonstrated that SVF-gel exhibited higher percentage volume maintenance than did Coleman's fat at the 12 months follow-up. Previous 3D research reported that the volume retention for facial augmentation with centrifuged fat was 43% at the 3 months follow-up. Wu et al. (1) reported that the volume retention rate if centrifuged fat for facial augmentation was 31% at the 6 month follow-up. Further, Guibert et al. (19) observed that the volume retention for facial malformations corrected with centrifuged fat was 36% at the 6 months follow-up. At the 12 months follow-up in this trial, the retention rates in the SVF-gel and Coleman's fat groups were 41.2% ± 8.4% and 32.6% ± 8.8%, respectively, which were lower than those of other studies. This difference could be due to the injection layer (20). Our findings for volume retention of temple augmentation with Coleman's fat at 12 months follow-up coincide with prior observations. Interestingly, we found that long-term assessment of transplanted SVF-gel identified a higher volumizing effect on temple augmentation than did Coleman's fat. Further, the patient satisfaction rate was higher in the SVF-gel group than that in Coleman's fat group at the 12 months follow-up.

By shifting through the syringes, large mature adipocytes were raptured and the free lipid was released and removed after centrifugation. SVF-gel may eliminate most of the lipid but leaves SVF cells and ECM behind (3). The high condensation of ASCs and adipose ECM in SVF-gel is thought to contribute to the improved survival of SVF-gel grafts by promoting angiogenesis and adipogenesis. Cell-assisted lipotransfer (CAL) technique, which added ASCs into autologous fat grafting and had enhanced the fat graft retention rate (21, 22). Clinical findings identified the advantage of the lipotransfer technique in enriching fat grafts with stem cells, improving long-term fat graft retention (23, 24). Compared with Coleman's fat, SVF-gel contains condensed ASCs, which are more likely to survive under physical stress and hypoxic and are the key for blood vessel regeneration after transplantation (25). Furthermore, SVF-gel contains ECM comprised of a collagen scaffold and scaffold-bound bioactive components. Adipose ECM creates a complex 3D bioactive microenvironment network, which can spontaneously induce adipose tissue regeneration *in vivo*. In contrast, survival of the peripheral region of the Coleman's fat grafts depends mainly on the infiltration of tissue fluid, the longer duration of inflammation may cause the central necrosis (4, 5, 26, 27). Therefore, the substantial biocomponent of adipose tissue would largely remain in SVF-gel, which could contribute to superior graft volume retention compared with conventional Coleman's fat grafting. Due to the higher retention rate and fewer postoperative complications, such as oil cysts and nodules, achieved after SVF-gel transplantation, SVF-gel is theoretically suitable for filling almost all soft tissue defection (4). However, the volume of SVF-gel prepared is less than 15% of the original Coleman's fat volume, a massive aspiration is required. Therefore, it is not suitable for breast augmentation with SVF-gel only or large volume filling in low-weight patients as of yet (3).

The present study is the first to report the effect of SVF-gel on temple contouring and long-term retention rates in comparison with Coleman's fat grafting. Considering that no standard for evaluating temple aesthetics exists, an evaluation system that combines temple contour by measuring forehead width and temporal peak in photographs, and temple augmentation by 3D laser scanning, was used in this study. This evaluation system is an easy and effective method to evaluate the outcome of temporal fat grafting.

The study has some limitations that should be acknowledged to avoid its overinterpretation. First, this was a single-center study with limited subjects, rather than a multicenter study with a large number of subjects. Second, 3D laser scanning is a relatively new tool in our department, necessitating data collection from more patients with a longer follow-up period.

## Conclusion

The present study demonstrated that SVF-gel is an effective, reproducible, and safe approach for temporal hollowing contouring and augmentation. We developed a new evaluation system combining temple contouring by measuring forehead width and temporal peak in photographs with temple augmentation by 3D laser scanning, which is an easy and effective method to evaluate the outcome of temporal fat grafting.

## Data Availability

The original contributions presented in the study are included in the article/Supplementary Material, further inquiries can be directed to the corresponding author/s.
