# Corrigendum: Contouring and augmentation of the temple using stromal vascular fraction gel grafting

**DOI:** 10.3389/fsurg.2022.1045744

**Published:** 2022-10-12

**Authors:** Yuchen Zhang, Jialiang Zou, Yi Yuan, Jianhua Gao, Xihang Chen

**Affiliations:** Department of Plastic and Reconstructive Surgery, Nanfang Hospital, Southern Medical University, Guangzhou, China

**Keywords:** SVF-gel, fat grafting, volume retention rate, 3D scanning, temple

A corrigendum on Contouring and augmentation of the temple using stromal vascular fraction gel grafting by Zhang Y, Zou J, Yuan Y, Gao J and Chen X. (2022) Front. Surg. 9: 893219. doi: 10.3389/fsurg.2022.893219

**Incorrect Funding**

In the published article, there was an error in the Funding statement. The funding statement for the Science and Technology Program of Guangzhou, China was displayed as “202201011577”. The correct statement is “the Science and Technology Program of Guangzhou, China (202201011571).”. The correct Funding statement appears below.

**Funding**

This work was supported by the National Natural Science Foundation of China (81871573, 81701920 and 81671931), the President Foundation of Nanfang Hospital (2019C010), the Science and Technology Program of Guangzhou, China (202201011571), and the China Postdoctoral Science Foundation (2020M672721).

The authors apologize for this error and state that this does not change the scientific conclusions of the article in any way. The original article has been updated.

