# Supplementary material for: Contouring and augmentation of the temple using stromal vascular fraction gel grafting
Source: Front Surg. 2022 Aug 16;9:893219. doi: 10.3389/fsurg.2022.893219 (PMC9428844; doi:10.3389/fsurg.2022.893219)
Supplement: Supplementary file 1 [file Data_Sheet_1.docx]

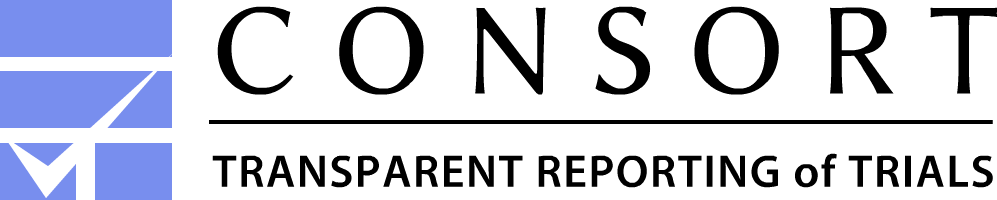


**CONSORT 2010 Flow Diagram**

Analyzed (n = 34)
 Excluded from analysis (n = 0)

Lost to follow-up (reasons given) (n = 2)

Discontinued intervention (n = 0)

Allocated to SVF-gel (n = 36)

 Received allocated intervention (n = 36)

 Did not receive allocated intervention (reasons given) (n = 0)

Allocated to conventional fat (n = 31)

 Received allocated intervention (n = 31 )

 Did not receive allocated intervention (reasons given) (n = 0 )

Randomization (n = 67)

Assessment of potential subjects for eligibility (n = 74)

## Enrollment

Excluded (n = 7)

  Did not meet inclusion criteria (n = 6)

  Declined to participate (n = 1)

  Other reasons (n = 0)

## Allocation

## Follow-up

Lost to follow-up (reasons given) (n = 1)

Discontinued intervention (n = 1)

## Analysis

Analyzed (n = 29)
 Excluded from analysis (n = 0)

SDC 1. Flow diagram of subject enrollment and outcomes.


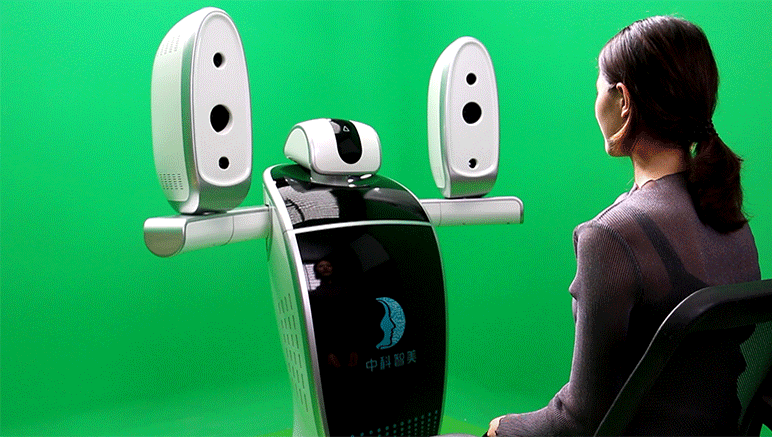


SDC 2. Schematic of the 3D imaging performed using a CASZM MVS-600.

|  |  | SVF-gel group  Mean (SD) | Coleman’s fat group  Mean (SD) | | *p-*value |
| --- | --- | --- | --- | --- | --- |
| Sex | Female | 30 | | 27 | 0.49 |
|  | Male | 4 | | 2 | 0.49 |
| Average age at screening, years | | 32.4(6.6)  range, 22–48 | | 33.3(7.4)  range, 22 – 49 | 0.61 |
| Average BMI at screening, kg/m^2^ | | 23.0(2.2)  range, 18.8–26.3 | | 23.5(3.0)  range, 18.4–27.5 | 0.45 |
| Average BMI at 12 months follow-up, kg/m^2^ | | 22.9(2.6)  range, 18.1–28.9 | | 23.1(3.5)  range, 18.3–29.2 | 0.79 |
| *p-*value | | 0.86 | | 0.64 |  |

SDC 3, Table. Subject demographics.

Values are expressed as mean (standard deviation). A two-tailed Student’s t-test was used to generate *p*-values.

BMI, body mass index.

|  |  | **SVF-gel group** | **Coleman’s fat group** | ***p-*value** |
| --- | --- | --- | --- | --- |
| **Donor sites** | Abdomen | 23 | 20 | 0.91 |
|  | Thighs | 11 | 9 | 0.91 |
| **Receiving area** | Temporal | 24 | 22 | 0.26 |
|  | Temporal and cheek | 10 | 7 | 0.26 |
| **Injection volume, ml** | | 5.5(1.0)  range, 3.2-7.7 | 5.9(1.4)  range, 3.2–9.8 | 0.06 |

SDC 4, Table. Fat harvesting and injection sites.

Values are expressed as mean (standard deviation). A Wilcoxon signed rank test was used to generate *p-*values.
